# Curcumin inhibits VEGF-mediated angiogenesis in human intestinal microvascular endothelial cells through COX-2 and MAPK inhibition

**DOI:** 10.1136/gut.2008.152496

**Published:** 2008-07-02

**Authors:** D G Binion, M F Otterson, P Rafiee

**Affiliations:** 1 Department of Medicine, Medical College of Wisconsin, Milwaukee, Wisconsin, USA; 2 Department of Surgery, Medical College of Wisconsin, Milwaukee, Wisconsin, USA

## Abstract

**Background:**

Angiogenesis, the growth of new blood vessels, is a critical homeostatic mechanism which regulates vascular populations in response to physiological requirements and pathophysiological demand, including chronic inflammation and cancer. The importance of angiogenesis in gastrointestinal chronic inflammation and cancer has been defined, as antiangiogenic therapy has demonstrated benefit in models of inflammatory bowel disease and colon cancer treatment. Curcumin is a natural product undergoing evaluation for the treatment of chronic inflammation, including inflammatory bowel disease (IBD). The effect of curcumin on human intestinal angiogenesis is not defined.

**Methods:**

The antiangiogenic effect of curcumin on in vitro angiogenesis was examined using primary cultures of human intestinal microvascular endothelial cells (HIMECs), stimulated with vascular endothelial growth factor (VEGF).

**Results:**

Curcumin inhibited proliferation, cell migration and tube formation in HIMECs induced by VEGF. Activation of HIMECs by VEGF resulted in enhanced expression of cyclo-oxygenase-2 (COX-2) mRNA, protein and prostaglandin E_2_ (PGE_2_) production. Pretreatment of HIMECs with 10 μM curcumin as well as 1 μM NS398, a selective inhibitor of COX-2, resulted in inhibition of COX-2 at the mRNA and protein level and PGE_2_ production. Similarly COX-2 expression in HIMECs was significantly inhibited by Jun N-terminal kinase (JNK; SP600125) and p38 mitogen-activated protein kinase (MAPK; SB203580) inhibitors and was reduced by p44/42 MAPK inhibitor (PD098059).

**Conclusions:**

Taken together, these data demonstrate an important role for COX-2 in the regulation of angiogenesis in HIMECs via MAPKs. Moreover, curcumin inhibits microvascular endothelial cell angiogenesis through inhibition of COX-2 expression and PGE_2_ production, suggesting that this natural product possesses antiangiogenic properties, which warrants further investigation as adjuvant treatment of IBD and cancer.

Vascular endothelial growth factor (VEGF) plays an essential role in endothelial proliferation and angiogenesis during embryonic development as well as periods of increased physiological, demand including the menstrual cycle, pregnancy and wound healing.^1^ ^2^ Enhanced expression of VEGF also occurs in disease conditions leading to pathological angiogenesis including chronic inflammation (ie, rheumatoid arthritis, psoriasis, inflammatory bowel disease (IBD)), diabetic retinopathy and adenocarcinoma.^3^ The importance of angiogenesis in disease processes has been demonstrated by the success of antiangiogenic therapeutic trials, which are approved for the treatment of advanced colorectal adenocarcinoma.^4^ VEGF plays a key role in cancer biology and contributes to tumour neovascularisation in response to the increased demand for delivery of nutrients and oxygen.^5^^–^7 In the setting of chronic inflammation, antiangiogenic therapy has shown beneficial effects in animal models of IBD (Crohn’s disease, ulcerative colitis)^8^ as well as open-label trials of the compound thalidomide in refractory Crohn’s disease.

The cyclo-oxygenase (COX) enzymes are involved in numerous physiological responses including inflammation, where they catalyse the synthesis of prostaglandins (PGs) from arachidonic acid. COX-1 is one of the two COX isoforms, and is responsible for maintaining normal physiological functions; it is expressed constitutively in most tissues. In contrast, COX-2 is an early response gene induced by growth factors, proinflammatory cytokines, tumour promoters and bacterial toxins.^9^^–^11 Inhibition of COX-2 by non-steroidal anti-inflammatory drugs (NSAIDs) results in inhibition of angiogenesis and downregulation of angiogenic factors such as VEGF and bFGF-2 (basic fibroblast growth factor).^12^^–^14 In human colorectal adenocarcinoma and other malignancies such as breast, cervical, prostate and lung tumours, increased COX-2 expression has been reported.^15^ ^16^ In mice, inhibition of COX-2 has been shown to protect against intestinal polyposis.^17^ The precise mechanisms whereby COX-2 contributes to tumourigenesis include effects on the epithelium, but additional effects on non-epithelial populations including the microvascular endothelium have also been suggested.^11^

Curcumin, the major yellow colouring pigment found in the household spice turmeric (*Curcuma longa* Linn, Zingiberaceae), has been used for centuries in food preparation as well as in Ayurvedic traditional medicine to treat inflammatory disorders.^18^ Curcumin has low toxicity and has been shown to benefit the treatment of chronic gut inflammation in animal models, as well as showing benefit in a randomised cross-over trial in the treatment of ulcerative colitis.^19^ Also, curcumin has been shown to have antineoplastic potential, inhibiting the development of chemically induced tumours of the oral cavity, skin, forestomach, duodenum and colon in rodents.^20^^–^23 The effect of curcumin on pathological angiogenesis associated with gastrointestinal disease processes has not been defined.

Our laboratory has focused investigation on the microvascular endothelial biology of the human gastrointestinal tract, utilising primary cultures of human intestinal microvascular endothelial cells (HIMECs). Previously, we have shown that VEGF leads to proliferation of HIMECs,^24^ triggering dephosphorylation, translocation and activation of NFAT (nuclear factor of activated T cells) in HIMECs.^25^ VEGF activates various signalling pathways such as phosphatidylinositol 3-kinase (PI3K)/Akt, protein kinase C (PKC) and mitogen-activated protein kinase (MAPK) cascades.^26^ However, the signalling pathways by which VEGF regulates COX expression in HIMECs are not fully characterised.

In the present study, we examined the effect of curcumin and MAPK inhibitors on COX-2 gene expression and angiogenesis in HIMECs following VEGF stimulation. We have shown that COX-2 plays an important role in VEGF-induced angiogenesis via MAPKs, and curcumin blocks both COX-2 expression and angiogenesis induced by VEGF. These results may provide a mechanistic understanding for the beneficial effects of curcumin in conditions including chronic inflammation and cancer, where pathological neovascularisation is associated with an enhanced expression of VEGF.

## MATERIALS AND METHODS

### Reagents

Endothelial cell growth supplement (ECGS) was from Upstate Cell Signaling Solutions (Temecula, California, USA). RPMI 1640 medium, fetal bovine serum (FBS), MCDB-131 medium and PSF (penicillin/streptomycin/fungizone) were obtained from Invitrogen (Carlsbad, California, USA). Human plasma fibronectin was purchased from Chemicon International (Temecula, California, USA). Porcine heparin was from Sigma Chemical Co. (St Louis, Missouri, USA). The recombinant human VEGF, antibodies to intercellular adhesion molecule 1 (ICAM-1), vascular cell adhesion molecule (VCAM) and E-selectin were purchased from R&D Systems (Minneapolis, Minnesota, USA). The COX-1/-2 antibodies were obtained from Santa Cruz Biotechnology (Santa Cruz, California, USA). The selective COX-2 inhibitor NS398 and carbacyclin (stable analogue of PGI_2_) were obtained from Cayman Chemical (Ann Arbor, Michigan, USA). Antibodies against phosphorylated and non-phosphorylated MAPK family members (p44/42 MAPK, p38 MAPK and JNK (Jun N-terminal kinase)) were from Cell Signaling Technology (Danvers, Massachusetts, USA). The MAPK inhibitors (PD098059, SB203580 and SP600125) were obtained from Calbiochem (La Jolla, California, USA). Immun-Star and all other electrophoresis reagents were from Bio-Rad (Hercules, California, USA). Oligonucleotides and primers were purchased from IDT (Integrated DNA Technologies, Coralville, Iowa, USA). Fluorescein-conjugated phalloidin was from Molecular Probes (Eugene, Oregon, USA). Unless otherwise indicated, all other chemicals used in this study were purchased from Sigma-Aldrich. All experiments were approved by the Institutional Review Board of the Medical College of Wisconsin.

### HIMEC isolation and culture

HIMECs were isolated and cultured as previously described.^24^ Experiments were performed on three independent HIMEC lines unless otherwise specified. All images displayed were a representative result of one of the three independent experiments.

### Activation and pharmacological modulation of HIMECs

HIMEC activation was achieved following VEGF (50 ng/ml) stimulation for specified time periods. Curcumin (10 μM), NS398 (1 μM), SB203580 (5 µM), PD098059 (10 µM) and SP600125 (10 µM) were used to determine the effect of these inhibitors on COX-2 expression and angiogenesis.

### Cell proliferation assay

A total of 3×10^4^ HIMECs per well were seeded onto fibronectin-coated 24-well plates, and proliferation assays were performed as previously described.^25^ After pretreatment with 10 μM curcumin and 1 μM NS398, 5 µM SB203580, 10 µM PD098059 and 10 µM SP600125 for 30 min at 37°C, cells were stimulated with VEGF (50 ng/ml) for 12, 24, 48 and 72 h, or left untreated. Then, cells were re-suspended and counted in a Coulter Counter (Coulter, Brea, California, USA). In parallel experiments, cell viability was assessed by trypan blue exclusion and was >95%. Each condition was assessed in triplicate.

Cellular DNA synthesis was assessed by [3H]thymidine uptake.^25^ HIMECs were pulsed with [3H]thymidine (1 µCi/ml; Amersham, Arlington Heights, Illinois, USA) and washed with 5% (v/v) trichloroacetic acid (2×) prior to fixation. Using 0.5 N NaOH, the DNA was precipitated, and supernatants were quantified in a beta counter. Each condition was assessed in triplicate.

### Microscopic wounding assay

To assess the effect of curcumin on HIMEC growth following angiogenic stimuli, a microscopic wounding assay was performed as described earlier.^25^ In brief, a HIMEC confluent monolayer was scraped along a straight line, and the remaining monolayer was then incubated with growth medium (without ECGS), and cells were pretreated for 30 min at 37°C with or without curcumin (10 µM) or NS398 (1 µM). Then, cells were stimulated by addition of VEGF (50 ng/ml) or left untreated. The migration of HIMECs across the demarcation line was monitored using an inverted microscope. At each time point (0, 24, 48 and 72 h), 10 random fields were counted in a blinded fashion using an ocular grid. Data were expressed as cells/mm^2^, and each condition was assessed in triplicate.

### Endothelial cell chemotaxis assay

HIMECs (3×10^4^ cells/cm^2^) were cultured on fibronectin-coated polycarbonate filters (8 μm pore size, BD Biosciences, Bedford, Massachusetts, USA) as previously described.^25^ After incubation in overnight medium containing 2% FBS, HIMECs were incubated with curcumin (0–20 μM) or NS398 (0–10 μM) for 2 h, and buffers containing VEGF (50 ng/ml) were poured into the lower compartment of the 12-well plates. When indicated, PGE_2_ (0.1–1 μM) was added to both upper and lower chambers. After overnight incubation, cell culture inserts were removed and the upper surface of the membrane was gently wiped to remove non-migrated cells. Filters were stained with DiffQuik (Baxter Scientific, McGraw, Illinois, USA), air-dried, and mounted onto glass slides. Migrated HIMECs adherent to the lower side of the membrane were counted (10 random high-power fields (40×) per condition in a blinded fashion). Cell viability was >95% as assessed by trypan blue exclusion. Each condition was assessed in triplicate, and data were expressed as a mean (SD).

### Matrigel in vitro tube formation assay

HIMEC tube formation was assessed using Matrigel, as described previously.^25^ Where indicated, the culture medium was supplemented with various inhibitors as above, while control wells contained no inhibitors. Using an inverted phase contrast microscope, HIMEC tube formation was assessed. Five high-power fields per condition were examined and experiments were repeated in three independent HIMEC cultures.

### ELISAs of PGE_2_ and 6-keto PGF_1α_ in HIMEC culture medium

Confluent HIMEC monolayers were assayed as previously described.^27^ Briefly, HIMECs were pretreated for 2 h with curcumin (0–20 μM) then stimulated with 50 ng/ml VEGF for 8 h. The concentration of PGE_2_ and 6-keto PGF_1α_ (the stable metabolite of PGI_2_) in the culture supernatant was determined using a commercial ELISA (Cayman Chemical). To determine COX isozymes which produce PGE_2_ and PGI_2_, HIMECs were pretreated with NS398 (0–10 μM), the COX-2-specific inhibitor, for 2 h then stimulated with 50 ng/ml VEGF for 8 h. The concentration of PGE_2_ and 6-keto PGF_1α_ was determined as described above. Experiments were carried out in triplicate, and results are shown as mean pg/ml (SD).

### RNA preparation and semi-quantitative reverse transcription–PCR (RT–PCR)

COX-1 and COX-2 mRNA expression were determined as described previously.^25^ PCR amplifications were performed as follows: 30 cycles for COX-1 (94°C for 1 min, 56°C for 1 min, 72°C for 1 min), 35 cycles for COX-2 (94°C for 1 min, 54°C for 1 min, 72°C for 1 min) and 25 cycles for β-actin (94°C for 1 min, 60°C for 1 min, 72°C for 1 min), using COX-1 forward (5′-TGC CCA GCT CCT GGC CCG CCG CT-3′) and reverse (5′-TTC AAA TGA GAT TGT GGG AAA ATT GTC-3′); COX-2 forward (5′- TCA AAT GAG ATT GTG GGA AAA TTG-3′) and reverse (5′-TCT AGT AGA GAC GGA CTC ATA GAA-3′); and β-actin forward (5′-CCA GAG CAA GAG AGG CAT CC-3′) and reverse (5′-CTG TGG TGG TGA AGC TGT AG-3′) specific primers, followed by final extension for 7 min at 72°C. The PCR products were visualised on 1.2% agarose gels stained with ethidium bromide. RNA solution without reverse transcription was used as negative control (no RT), and β-actin served as an internal control.

### Western blot analysis

Confluent HIMEC monolayers in 35 mm culture dishes (one dish per condition) were pretreated with various inhibitors for 30 min or left untreated before VEGF activation (50 ng/ml) for different time periods. Sodium dodecylsulfate–polyacrylamide gel electrophoresis (SDS–PAGE) and western blot analysis were performed using antibodies to COX-1, COX-2 and MAPKs as described previously.^25^ Detection was by secondary antibody coupled to horseradish peroxidase (HRP) and Immun-Star (Bio-Rad).

### Immunofluorescence staining

HIMEC monolayers were grown on coverslips to 80% confluence. Following curcumin or NS398 treatment and VEGF activation, monolayers were rinsed once in phosphate-buffered saline (PBS), fixed with cold methanol for 30 min and blocked with 5% bovine serum albumin in PBS with Ca^2+^ and Mg^2+^ for 60 min. Using COX-2 antibody, followed by a fluorescein isothiocyanate (FITC)-conjugated secondary antibody (Santa Cruz Biotechnology), the effect of curcumin on COX-2 expression was visualised. Coverslips were mounted on Superfrost slides (Fisher Scientfic) with Prolong Antifade mounting medium (Invitrogen) and visualised using a fluorescence microscope (Olympus BX-40) and a Leica DFC 300FX camera.

### Assessment of cell adhesion molecule (CAM) surface expression by tumour necrosis factor α (TNFα)/lipopolysaccharide (LPS) in HIMECs

CAM surface expression in HIMECs was assessed following TNFα/LPS (100 U/ml TNFα, 1 μg/ml LPS) activation with or without 10 μM curcumin pretreatment using radioactive immunoassay (RIA) and flow cytometry (fluorescence activated cell sorting (FACS)) as described previously.^28^ ^29^

### Leucocyte adhesion assay on HIMECs

Leucocyte–HIMEC interaction with or without 10 μM curcumin pretreatment was assessed using a low shear stress flow adhesion assay and U-937 cells as described previously.^28^ ^29^

## RESULTS

### VEGF induced increased COX-2 expression in HIMECs

**Figure 1:**
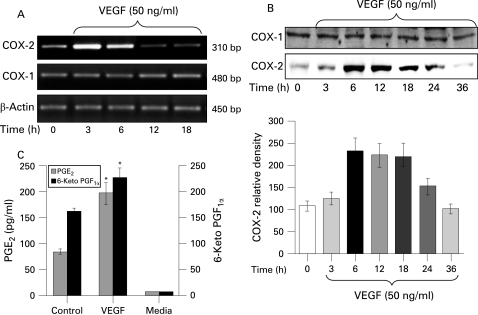
Effect of vascular cell adhesion factor (VEGF) on cyclo-oxygenase-1 (COX-1) and COX-2 mRNA and protein expression in human intestinal microvascular endothelial cells (HIMECs). (A) Semi-quantitative reverse transcription–PCR of COX mRNA in HIMECs demonstrates that COX-1 mRNA, but not COX-2, was constitutively expressed in HIMECs. VEGF (50 ng/ml) stimulation of HIMECs resulted in marked upregulation of COX-2 gene expression by 3 h, whereas COX-1 was unaffected. β-Actin was used as an internal loading control. (B) Western blot analysis demonstrates that VEGF enhanced COX-2 protein expression in HIMECs by 6 h, which persisted until 24 h, declining by 36 h. COX-1 protein expression was not affected by VEGF. (C) The results of ELISA for prostaglandin E_2_ (PGE_2_) and 6-keto PGF_1α_ in HIMEC culture supernatants demonstrate that VEGF stimulation significantly increased both PGE_2_ and 6-keto PGF_1α_ production in HIMECs. Data are expressed as pg/ml PG production (SD). *p<0.05 compared with control.

Activation of HIMECs with 50 ng/ml VEGF resulted in enhanced COX-2 expression at the mRNA level as detected by RT–PCR using COX-2 primers. Enhanced COX-2 mRNA expression was time dependent, increasing by 3 h, and declining by 18 h (fig 1A). β-Actin gene expression was used as an internal control in these experiments. In marked contrast, levels of COX-1 mRNA expression remained unchanged following VEGF stimulation of HIMECs (Fig. 1A).

Next we determined the effect of VEGF on COX-2 protein expression by western blotting using a specific COX-2 antibody. VEGF strongly and time dependently (6–24 h) enhanced the expression of COX-2 protein in HIMECs (fig 1B). There was no detectable change in the level of COX-1 protein after VEGF stimulation (fig 1B).

Corresponding to the effect of VEGF on COX-2 mRNA and protein expression, the ELISA results from the HIMEC culture supernatant demonstrated that VEGF (50 ng/ml) stimulation for 12 h significantly increased PGE_2_ and 6-keto PGF_1α_ production (fig 1C).

### Curcumin inhibits COX-2 expression and PGE_2_ production in HIMECs

**Figure 2:**
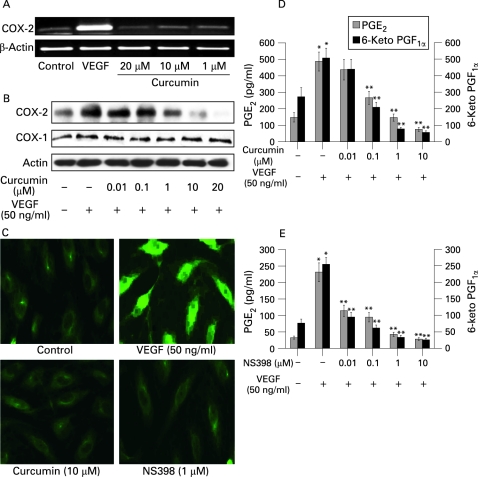
Curcumin inhibits cyclo-oxygenase-2 (COX-2) expression in human intestinal microvascular endothelial cells (HIMECs). (A) Pretreatment of HIMECs with curcumin abolished vascular endothelial growth factor (VEGF) induction of COX-2 mRNA expression in a dose-dependent fashion. (B) Consistent with the gene expression data, curcumin pretreatment of HIMECs inhibited COX-2 protein expression at 1 µM, increasing at 10 µM, while 20 µM curcumin completely abolished COX-2 expression following VEGF activation. (C) Immunofluorescence staining of HIMECs pretreated with either 10 μM curcumin or 1 μM NS398 demonstrated inhibition of COX-2 expression following VEGF activation. (D) Curcumin inhibited both prostaglandin E_2_ (PGE_2_) and 6-keto PGF_1α_ production in HIMECs in a dose-dependent manner. Prostanoid species were assessed from HIMEC culture media using ELISA. Data were expressed as pg/ml PG production (SD). *p<0.05 compared with no stimulation; **p<0.05 compared with VEGF stimulation. (E) PGE_2_ and 6-keto PGF_1α_ production were also completely inhibited by pre-treatment with 1 μM NS398. Data were expressed as pg/ml PG production (SD). *p<0.05 compared with no stimulation; **p<0.05 compared with VEGF stimulation.

Next we examined the effect of curcumin on COX-2 gene and protein expression. Pretreatment of HIMECs with curcumin abolished VEGF induction of COX-2 mRNA expression in a dose-dependent fashion (fig 2A). Consistent with the gene expression data, curcumin pretreatment of HIMECs inhibited COX-2 protein expression at 1 µM, with increasing effect at 10 µM curcumin, while 20 µM curcumin completely abolished COX-2 expression following VEGF activation (fig 2B). The selective inhibitor of COX-2, NS398 (1 µM), abolished COX-2 mRNA and protein expression in HIMECs following VEGF activation (not shown). In addition, immunofluorescence staining of HIMECs pretreated with either 10 μM curcumin or 1 μM NS398 demonstrated inhibition of COX-2 expression following VEGF activation (fig 2C).

Corresponding with the effect on COX-2 expression, curcumin inhibited both PGE_2_ and 6-keto PGF_1α_ production in a dose-dependent fashion as determined by ELISA measurement of HIMEC culture media (fig 2D). PGE_2_ and 6-keto PGF_1α_ production were completely inhibited by 1 μM NS398 (pre-treatment), indicating that production of this prostanoid is dependent on COX-2 activity in HIMECs (fig 2E).

### Curcumin inhibits HIMEC growth, proliferation, migration and tube formation

Angiogenesis involves multiple events in endothelial cells, including cell migration, proliferation and tube formation. Previously, we have demonstrated that VEGF activation of HIMECs resulted in cell migration, proliferation, tube and stress fibre formation.^25^ To determine the antiangiogenic potential of curcumin and its potential mechanism of action through inhibition of COX-2 expression, in vitro angiogenesis assays following VEGF stimulation measuring growth, proliferation, transmigration and tube formation were performed in HIMECs, using NS398 as a specific COX-2 inhibitor.

**Figure 3:**
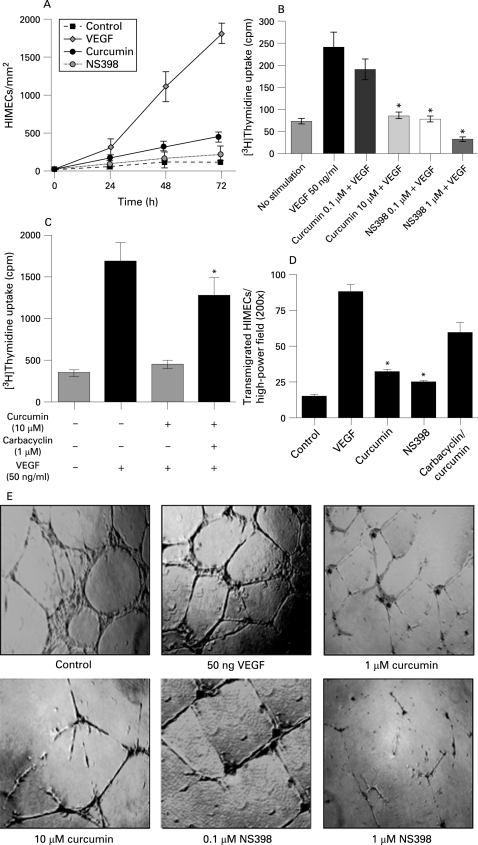
Curcumin inhibits growth, proliferation, migration and tube formation in human intestinal microvascular endothelial cells (HIMECs). (A) Potent angiogenic effect of vascular endothelial growth factor (VEGF) compared with no stimulation in HIMECs. Curcumin inhibited HIMEC growth at rates almost similar to those of control cells. The cyclo-oxygenase-2 (COX-2) inhibitor, NS398 was a potent inhibitor of cell growth. (B) Cellular DNA synthesis was assessed by measuring [3H]thymidine uptake. [3H]Thymidine uptake was significantly increased after VEGF stimulation for 15 h and was inhibited by curcumin pretreatment for 30 min in a dose-dependent manner. Assays were done in triplicate and the data are shown as mean cpm (SD). *p<0.05 compared with VEGF-stimulated HIMEC cultures. (C) The inhibitory effect of curcumin on [3H]thymidine uptake was reversed by addition of carbacyclin (a prostaglandin I_2_ (PGI_2_) analogue). *p<0.05 compared with curcumin-treated HIMECs with no exogenous PG. (D) The number of HIMECs transmigrated through the filter was increased by VEGF stimulation; curcumin pretreatment of HIMECs significantly inhibited HIMEC transmigration, which was reversed by 1 μM carbacyclin. At least 15 random high-power fields (×200) per condition were counted and data were expressed as mean (SD). *p<0.05 compared with curcumin pretreatment. (E) Phase-contrast photomicrograph demonstrates the endothelial in vitro tube formation on Matrigel; the formation of capillary-like structures was inhibited by curcumin pretreatment (×40).

Initial experiments were performed evaluating endothelial growth in a wounded monolayer, with cell expansion across a leading edge.^30^ Figure 3A demonstrates a potent angiogenic effect of VEGF compared with control cells. Curcumin-pretreated HIMEC monolayers were unresponsive to VEGF, and grew at rates almost similar to those of untreated cells. Curcumin demonstrated no toxicity at the dosages used in this study. The COX-2 inhibitor NS398 was a potent inhibitor of endothelial cell growth.

We next performed in vitro growth studies to assess the effect of curcumin on HIMEC growth. Cell cycle re-entry and DNA replication in endothelial cells is a requisite step in angiogenesis. Proliferation was determined by measuring both [3H]thymidine uptake and cell enumeration using a Coulter counter. [3H]Thymidine uptake was significantly increased after VEGF treatment, and VEGF stimulation for 18 h also significantly increased cell number. Curcumin pretreatment of HIMECs following VEGF activation resulted in inhibition of [3H]thymidine uptake and reduced cell number significantly (fig 3B). The cellular viability remained >98% in all groups (data not shown). The inhibitory effect of curcumin on HIMEC [3H]thymidine uptake was reversed by addition of the PGI_2_ analogue carbacyclin (fig 3C). Proliferation of HIMECs in response to VEGF was completely inhibited by both curcumin and the COX-2 inhibitor NS398.

Pretreatment of HIMECs with either curcumin or the COX-2-specific inhibitor NS398 resulted in inhibition of endothelial transmigration and tube formation. Curcumin pretreatment of HIMECs inhibited VEGF-induced transmigration, which was similar to the effect of the COX-2-specific inhibitor NS398 (fig 3D). The endothelial in vitro tube formation assay using Matrigel was performed in the absence and presence of curcumin or NS398 following VEGF activation. The number of endothelial tubes formed in Matrigel was significantly inhibited by both curcumin and NS398 (fig. 3E). The inhibitory effect of curcumin on endothelial tube formation was reversed by carbacyclin (not shown). These results indicate that inhibition of COX-2 activity is linked to impaired HIMEC angiogenesis in vitro. Finally, curcumin and NS398 both exerted a potent inhibitory effect on HIMECs, decreasing prostanoid production as demonstrated above (fig 2E, F).

### Pharmacological modulation of HIMECs

**Figure 4:**
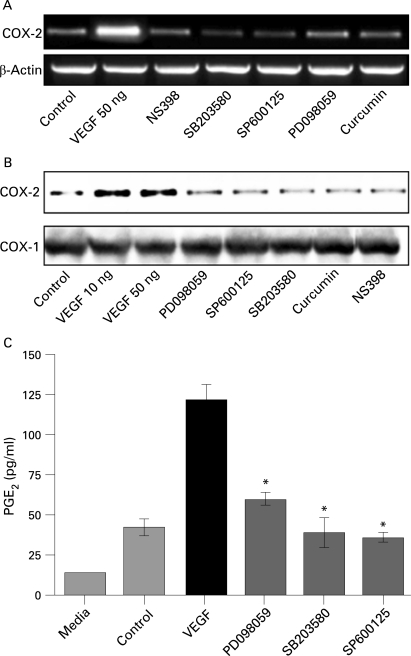
Modulation of cyclo-oxygenase-2 (COX-2) expression in human intestinal microvascular endothelial cells (HIMECs) by mitogen-activated protein kinase (MAPK) inhibitors. (A and B) Inhibition of the MAPK pathways resulted in downregulation and suppression of COX-2 mRNA and protein. Pretreatment of HIMECs with either 10 µM PD098059 (p44/42 MAPK), 5 µM SB203580 (p38 MAPK) or 10 µM SP600125 (Jun N-terminal kinase) significantly inhibited vascular endothelial growth factor (VEGF)-induced COX-2 mRNA and protein expression. (C) All three MAPK inhibitors inhibited prostaglandin E_2_ (PGE_2_) production as determined by ELISA in HIMEC culture media. *p <0.05 for inhibitors vs VEGF.

Previously we have shown that VEGF stimulation of HIMECs leads to a marked phosphorylation and activation of all three MAPK family members (p44/42 MAPK, stress-activated protein kinase (SAPK)/JNK and p38 MAPK).^25^ To investigate whether MAPK pathways play a role in VEGF induction of COX-2, HIMECs were pretreated with specific MAPK inhibitors then activated with VEGF. Our findings show that inhibition of the MAPK pathways resulted in downregulation and suppression of COX-2 expression at both the mRNA and protein levels. Inhibition of p44/42 MAPK by PD098059 (10 µM) inhibited COX-2 expression. Similarly, pretreatment of HIMECs with either SB203580 (5 µM) or SP600125 (10 µM), selective inhibitors of p38 MAPK and JNK, respectively, also significantly inhibited VEGF-induced COX-2 expression at both the mRNA and protein levels (fig 4A, B). Corresponding to the effect on COX-2 expression, all three MAPK inhibitors exerted an inhibitory effect on PGE_2_ production as determined by ELISA in HIMEC culture media (fig. 4C). These data suggest that all MAPK members are involved in upregulation of COX-2 expression by VEGF in HIMECs. In marked contrast, MAPK inhibitors did not affect COX-1 expression.

### Effect of curcumin on MAPK activation in HIMECs

**Figure 5:**
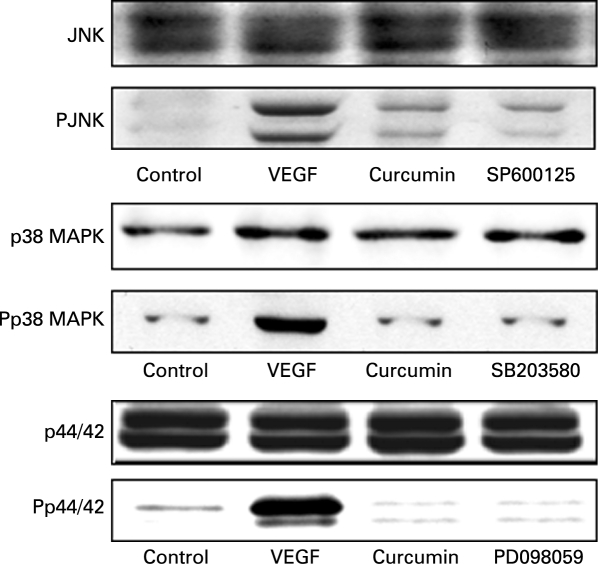
Curcumin inhibits the activation of mitogen-activated protein kinases (MAPKs) in vascular endothelial growth factor (VEGF)-activated human intestinal microvascular endothelial cells (HIMECs). Pretreatment of HIMECs with 10 µM curcumin following VEGF activation resulted in inhibition of p44/42 MAPK, p38 MAPK and Jun N-terminal kinase (JNK) phosphorylation.

Upregulation of COX-2 expression by VEGF via MAPK-dependent pathways has been shown in human umbilical vein endothelial cells.^13^ However, it is not known whether curcumin modulates the expression of COX-2 by inhibition of these MAPK pathways in VEGF-activated microvascular endothelial cells, specifically HIMECs. Thus, we examined the effect of curcumin on VEGF-induced activation of p44/42 MAPK, p38 MAPK and JNK in HIMECs. Previously we have shown that VEGF activation of HIMECs would lead to phosphorylation and activation of all three kinases.^25^ To confirm whether curcumin inhibits the activation of MAPKs in VEGF-activated HIMECs, these cells were pretreated with curcumin (10 µM) and were then stimulated by VEGF. As shown in fig 5, phosphorylation of p44/42 MAPK, p38 MAPK and JNK by VEGF was significantly decreased by curcumin pretreatment of HIMECs. These results indicate that curcumin attenuates VEGF-induced COX-2 expression through inhibition of MAPK pathways.

### Effect of curcumin on CAM expression in HIMECs following TNFα/LPS activation

**Figure 6:**
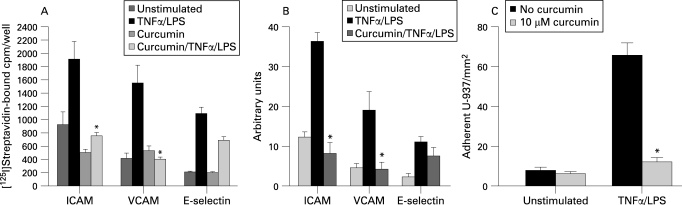
Effect of curcumin on cell adhesion molecule (CAM) expression and leucocyte binding in human intestinal microvascular endothelial cells (HIMECs). (A) Radioimmunoassay analysis demonstrates that curcumin pretreatment of HIMECs followed by tumour necrosis factor α (TNFα)/lipopolysaccharide (LPS) activation completely inhibited intercellular adhesion molecule 1 (ICAM-1) and vascular cell adhesion molecule (VCAM) expression but only partially reduced E-selectin. *p <0.05. (B) Fluorescence activated cell sorting analysis of HIMECs demonstrates the similar inhibitory effect of curcumin on CAM expression. Data are expressed as mean (SD) from triplicate wells. *p <0.05. (C) Low-shear stress flow adhesion assay demonstrates that curcumin pretreatment of HIMECs resulted in inhibition of U-937 leucocyte adhesion. *p <0.05.

CAM surface expression by endothelial cells is regulated by cytokines and inflammatory activators.^31^ ^32^ Previously, we have shown that the CAM expression in HIMECs was enhanced in response to dual inflammatory stimulation with TNFα/LPS.^28^ ^29^ Here, we examined the effect of curcumin pretreatment on CAM expression and leucocyte binding in HIMECs following TNFα/LPS activation. Figure 6A, using cell surface RIA, demonstrates that curcumin effectively inhibited the ICAM-1 and VCAM surface expression in response to TNFα/LPS activation and partially reduced E-selectin expression. Similar results were obtained from FACS analysis (fig 6B).

Next we performed a low-shear stress flow adhesion assay. As shown in fig 6C, curcumin (10 μM) pretreatment of HIMECs inhibited the adhesion of U-937 cells to TNFα/LPS-activated HIMECs.

## DISCUSSION

In this study, we demonstrate that curcumin exerts potent effects on the angiogenic properties of microvascular endothelial cells isolated from the human intestine, inhibiting multiple stages in the angiogenic process. We have demonstrated that (1) COX-2 induction as well as prostanoid production induced by VEGF were blocked by curcumin; (2) VEGF-induced growth, proliferation, transmigration and tube formation in HIMECs, an in vitro strategy for modelling angiogenesis, were also inhibited by curcumin; (3) the COX-2-specific inhibitor NS398, inhibited HIMEC growth, proliferation, transmigration and tube formation induced by VEGF; and (4) MAPK family members are involved in VEGF-induced upregulation of COX-2 and PGE_2_ production in HIMECs.

The cyclo-oxygenase enzymes COX-1 and COX-2 have been shown to play an important role in the regulation of angiogenesis.^33^ These enzymes catalyse the conversion of arachidonic acid to PGH_2_, the first step in the biosynthesis of the PGs thromboxane and prostacyclin.^34^ In endothelial cells, COX-1 is constitutively expressed, whereas COX-2 is inducible in response to various activators such as mitogens, hormones and inflammatory cytokines.^35^

Our findings suggest that curcumin exerts an inhibitory effect on microvascular endothelial growth, proliferation, migration and tube formation, and suppresses angiogenesis by inhibiting COX-2 expression and PG production. These data suggest that the inhibition of in vitro angiogenesis or reduced PG production will result from the inhibitory effect of curcumin on VEGF-induced HIMEC activation. Previously we have demonstrated that VEGF induced COX-2 expression, and COX-derived prostanoid production plays an important role in HIMEC angiogenesis.^27^ In the present study, we demonstrate that curcumin inhibits the angiogenic effect of VEGF-induced COX-2 mRNA and protein expression as well as PGE_2_ production in HIMECs. The inhibitory effect of curcumin on TNFα/LPS-induced COX-2 expression in HIMECs (data not shown) indicates that regardless of the type of induction, curcumin is a potent inhibitor of COX-2 expression and prostanoid production in endothelial cells. COX-2 expression and prostanoid production have been implicated as important mechanisms in angiogenesis during tumour growth, as COX-2 is upregulated in the microvasculature surrounding tumours,^36^ and inhibition of COX activity by NSAIDs has been shown to reduce angiogenesis and tumour growth both in vivo and in vitro.^37^ ^38^ The predominant role of endothelial COX-2 versus COX-1 in angiogenesis remains controversial.^33^ In the present study, we demonstrate that the COX-2 inhibitor NS398 impaired in vitro angiogenesis in HIMECs, supporting the idea that COX-2-derived prostanoids play a central role in angiogenesis. The fact that carbacyclin reversed the inhibition of [3H]thymidine uptake, cell migration and tube formation by curcumin also implicates the central role of COX-2 in HIMEC angiogenesis. Therefore, the inhibitory effect of curcumin could in part be the result of suppression of PGI_2_ formation through inhibition of COX-2 expression. In addition to the effect of curcumin on COX-2 expression, curcumin also affects expression of several genes associated with cell growth and/or apoptosis (eg, Bcl2, Bax, caspase and PI3K/Akt). Curcumin inhibition of endothelial nitric oxide synthase (eNOS) expression in endothelial cells has been shown to contribute to impaired endothelial tube formation.^39^ Taken together, our results suggest that the antiangiogenic activity of curcumin involves modulation of multiple pathways in endothelial cells.

The importance of antiangiogenic agents in the treatment of cancer is well established, and their role in the treatment of chronic inflammatory disorders is gaining momentum. Work in rheumatoid arthritis as well as human IBD has demonstrated a central role for angiogenesis in the pathophysiology of these chronic inflammatory diseases. Use of novel antiangiogenic agents in the treatment of animal models of IBD has also shown benefit. In experiments by Danese *et al*^8^ using ATN-161, a peptide inhibitor of the proangiogenic α_v_β_3_/α_5_β_1_ integrins, a significant therapeutic effect was shown in interleukin 10 (IL10) knockout mice. The IL10 knockout mouse is felt to be an established model of chronic inflammatory injury and remodelling in the gastrointestinal tract, and animals treated with this antiangiogenic compound showed improvement in their disease activity index, an improved histological colitis score with a parallel decrease in intestinal microvessel density.

To date, the use of antiangiogenic agents in the treatment of human IBD has undergone limited exploration. The prototypical antiangiogenic agent thalidomide has been shown to benefit patients with refractory Crohn’s disease, but is fraught with problems, including its terrible legacy of causing severe birth defects during its early clinical use as well as unacceptable rates of neuropathy as an adverse reaction when employed on a long-term basis.^40^ ^41^ Two open-label pilot studies of low-dose thalidomide in chronically active, steroid-dependent Crohn’s disease showed benefit, but did not demonstrate whether an antiangiogenic effect was present in the responding patients.^42^ The potential for selective and non-selective COX antagonists as antiangiogenic agents for the treatment of IBD is also problematic, as these agents will typically worsen bowel injury in animal models of the disease and have also been linked to clinical deterioration in IBD patients.^43^^–^46 Therefore, the addition of curcumin, as an antiangiogenic agent which does not have the potential for clinical adverse side effects, may prove to be of significant benefit. At present, the majority of agents used for the treatment of IBD are felt to function through the inhibition of inflammatory mechanisms, or specifically blocking inflammatory cytokines, and the addition of an antiangiogenic compound may exert unique therapeutic benefit.

The use of antiangiogenic agents in the treatment of gastrointestinal malignancies has emerged as the standard of care for metastatic lesions.^47^ The anti-VEGF antibodies bevacizumab and cetuximab have proved successful in clinical trials, and optimal regimens for the use of these agents in patients with metastatic colorectal adenocarcinoma are being defined.^48^ The potential for “cocktails” of antiangiogenic agents which may target multiple mechanisms in the angiogenic process has also been shown. In the case of mesenteric desmoids, a benign tumour with no standard treatment regimen, our group has shown that combination antiangiogenic therapy with toremifene and interferon α2b was effective in causing regression of these lesions.^49^ Therefore, the potential for the addition of curcumin in combination with other antiangiogenic strategies warrants evaluation.

In summary, our present study indicates that curcumin is a potent inhibitor of angiogenesis in HIMECs in vitro. Curcumin appears to exert its antiangiogenic effect through inhibition of COX-2 expression and prostanoid production. Given the importance of angiogenesis and tumour neovascularisation in cancer progression, our data also suggest that the anticancer effects of curcumin may also involve direct effects on local microvascular populations. Future clinical studies evaluating the long-term benefit of curcumin as an antiangiogenic agent in the treatment of chronic gut inflammation and bowel cancers are warranted.
